# Efficacy and Safety of Glucagon-Like Peptide-1 (GLP-1) Receptor Agonists for Spinal Fusion Outcomes: A Comprehensive Meta-Analysis

**DOI:** 10.7759/cureus.93065

**Published:** 2025-09-23

**Authors:** Mohamed Zahed, Mahmoud Elmesalmi, Khaled F Al-Kharouf, Sara E Elbahnasawy, Ziad El Menawy, Salam Elhanash, Mahmoud Odeh, Nour Elnaggar, Mohamed Hesham Gamal, Mahmoud M Elhady

**Affiliations:** 1 Orthopaedics, John Radcliffe Hospital, Oxford University Hospitals NHS Foundation Trust, Oxford, GBR; 2 Trauma and Orthopaedics, St George’s University Hospitals NHS Foundation Trust, London, GBR; 3 Emergency Medicine, University Hospital Southampton NHS Foundation Trust, Southampton, GBR; 4 Diagnostic Radiology, Menoufia University Hospital, Menoufia, EGY; 5 Trauma and Orthopaedics, University Hospital of Wales, Cardiff, GBR; 6 Paediatric Burns and Plastic Surgery, Royal Manchester Children’s Hospital, Manchester, GBR; 7 Trauma and Orthopaedics, Cardiff and Vale University Health Board, Cardiff, GBR; 8 Trauma and Orthopaedics, Faculty of Medicine, Zagazig University, Zagazig, EGY; 9 Pharmacy, Benha University Hospital, Benha, EGY; 10 Pharmacology and Therapeutics, Faculty of Pharmacy, Tanta university, Tanta, EGY; 11 Orthopaedics, Faculty of Medicine, Benha University, Benha, EGY

**Keywords:** cervical fusion, glp-1 receptor agonists, lumbar fusion, meta-analysis, orthopedic surgery, pseudoarthrosis, spinal fusion, systematic review

## Abstract

Spinal fusion is a widely performed surgical procedure for treating various spinal disorders, with lumbar fusion showing remarkably rapid growth worldwide. Despite positive outcomes after the procedure, it carries significant complications, most notably pseudarthrosis. Compromised blood supply is a key factor disrupting normal bone fusion, making optimal vascularization crucial for successful outcomes. Glucagon-like peptide-1 (GLP-1) receptor agonists, primarily used for diabetes management, demonstrate promising effects including enhanced glycemic control, improved vascular endothelial function, and direct enhancement of osteoblastic cell activity through GLP-1 receptors on bone precursor cells. Theoretically, GLP-1 receptor agonists should be beneficial for optimizing spinal fusion outcomes. We aim to systematically review and analyze the current evidence on the efficacy and safety of GLP-1 receptor agonists in promoting bone fusion and reducing complications in patients undergoing spinal fusion surgery.

We conducted a comprehensive systematic review following Cochrane guidelines. We searched PubMed, Web of Science, Scopus, Embase, and Cochrane Library for studies examining GLP-1 receptor agonists in spinal fusion procedures. We used the Newcastle-Ottawa Scale for the quality assessment of the included studies. We conducted a statistical analysis using RevMan 5.4 with risk ratios for dichotomous outcomes.

In total, 11 studies with a total of 14,344 participants were analyzed. GLP-1 receptor agonists significantly reduced pseudoarthrosis at six months (risk ratio (RR) = 0.63, 95% confidence interval (CI) = 0.54-0.74) and 12 months (RR = 0.64, 95% CI = 0.57-0.72), and significantly increased acute kidney injury (RR = 1.30, 95% CI = 1.03-1.65). No significant differences were observed for pseudoarthrosis at 24 months (RR = 1.03, 95% CI = 0.53-2.03), readmission rates (RR = 0.85, 95% CI = 0.48-1.51), cerebrovascular accidents (RR = 1.01, 95% CI = 0.63-1.62), and deep vein thrombosis (RR = 1.16, 95% CI = 0.78-1.72). Additionally, no significant reoperations or adverse effects were found. We also performed a subgroup analysis considering the diabetic stage, which showed valuable insights.

GLP-1 receptor agonists showed promising results in reducing pseudoarthrosis at short- to medium-term follow-up, indicating potential therapeutic benefits in bone healing applications. However, the increased risk of acute kidney injury suggests the need for careful patient monitoring and risk stratification. The lack of sustained benefit at 24 months and significant heterogeneity observed in several outcomes indicate that further investigation is warranted. Future research should focus on conducting larger, well-designed randomized controlled trials with standardized outcome definitions, longer follow-up periods, and comprehensive safety monitoring to establish optimal dosing protocols and patient selection criteria for GLP-1 receptor agonist therapy in orthopedic applications.

## Introduction and background

Spinal fusion is one of the most common surgical procedures worldwide used for various spinal disorders [1]. The operation permanently joins two or more vertebrae in the spine to cure tumors, trauma, or any other degenerative diseases or deformities [2]. Spinal fusion can be done in different areas of the spine, with the lumbar spinal fusion showing a huge increase and growth in use worldwide, with further increase expected in the future [3,4].

The growing incidence of spinal fusion surgery may be due to the positive outcomes for patients, especially the improvement in the quality of life with a high reduction of pain and restoration of lost nerve function [5]. Despite these benefits, the procedure is associated with several complications [6]. These complications include pseudarthrosis, which is considered a high burden after the operation [7]. Pseudarthrosis is characterized by the absence of solid bony fusion at 12 months following surgery, with reported incidence rates varying from 5% to 35% across different patient populations, with multi-level fusions [8,9]. Additionally, hardware-related failures, degeneration of levels above or below fusion, surgical site infections, and thromboembolic events can also occur after the procedure [6]. While pseudarthrosis has multifactorial origins, compromised blood supply represents a key mechanism disrupting normal bone fusion [10]. Optimal vascularization at the fusion site is fundamental for delivering the nutrients and providing the metabolic environment necessary for bone regeneration and remodeling [10].

While glucagon-like peptide-1 (GLP-1) receptor agonists have not been previously explicitly investigated in spinal fusion procedures, these therapeutic agents demonstrate beneficial effects that could potentially influence fusion outcomes. GLP-1 agonists have been shown to enhance glycemic control through hemoglobin A1c (HbA1c) reduction as the first use for diabetic patients [11]. Additionally, these drugs have been shown to improve the vascular endothelial function, which collectively contributes to better microvascular perfusion and reduced diabetic complications [12]. Similar to other medications that enhance tissue blood supply and cellular metabolism, the presence of GLP-1 receptors on osteoblastic precursor cells suggests a direct bone-related mechanism, with in vitro evidence demonstrating that GLP-1 agonist treatment enhances the survival and function of these bone-forming cells [13]. This dual action of improving both systemic vascular supply and local bone cell activity positions GLP-1 agonists among therapeutic agents that could theoretically optimize the biological environment necessary for successful spinal fusion.

We aim to systematically review and analyze the current evidence on the efficacy and safety of GLP-1 receptor agonists in promoting bone fusion and reducing complications in patients undergoing spinal fusion surgery.

## Review

Methodology

The research methodology followed established guidelines from the Cochrane handbook for systematic reviews of interventions [14]. Manuscript preparation adhered to the Preferred Reporting Items for Systematic Reviews and Meta-Analyses (PRISMA) statement requirements [15].

Search Strategy

A comprehensive systematic literature search was performed across multiple electronic databases to identify studies examining the role of GLP-1 receptor agonists in spinal fusion procedures. The databases included PubMed, Web of Science (WOS), Scopus, Embase, and Cochrane Library. The search strategy employed a combination of controlled vocabulary (MeSH terms) and free-text keywords related to “GLP-1 receptor agonists” and “spinal fusion.” Database-specific search terminology is presented in the Appendices.

Study Selection

After conducting thorough database searches, all identified records were imported into EndNote Software Version X-9. Once duplicate entries were removed, the remaining records underwent a two-phase screening approach: first, an initial review of titles and abstracts, followed by a detailed examination of complete texts. In the initial phase, two separate researchers independently evaluated the titles and abstracts of all records using predetermined inclusion and exclusion criteria. Any disagreements between the reviewers were resolved through joint discussion and consensus-building.

We followed the predefined inclusion criteria as studies were included if they met the following inclusion criteria: participants: adult patients (≥18 years) undergoing spinal fusion surgery at any part of the spine; intervention: use of GLP-1 receptor agonists (semaglutide, liraglutide, dulaglutide, exenatide, lixisenatide, albiglutide, tirzepatide, or other incretin mimetics) perioperatively or during the postoperative period; comparison: control groups receiving placebo, standard care, or alternative treatments; outcomes: acute kidney injury, cardiovascular accidents, deep vein thrombosis (DVT), myocardial infarction, pneumonia, pseudoarthrosis, readmission rate, reoperation rate, infection, and surgical transfusion; study design: randomized controlled trials (RCTs), non-randomized trials, and observational studies. Studies were excluded if they were animal studies or in vitro research, published in non-English languages, did not report GLP-1 receptor agonist use, or did not involve spinal fusion procedures. Any conference abstracts, editorials, letters, or review articles without original or insufficient data for meta-analysis extraction were also excluded. We also excluded any duplications in population or involved overlapping patient populations, or focused solely on non-spinal orthopedic procedures.

Data Extraction

Independent data collection was conducted by two reviewers employing a pre-piloted, systematic extraction framework. Reviewer conflicts were managed through consensus building or third-reviewer consultation. Information gathered comprised study descriptors (research team, publication year, design approach, cohort size, and follow-up length), participant demographics (age characteristics, sex composition, diabetes mellitus status, coexisting conditions including hypertension, obesity, and smoking history), intervention parameters (GLP-1 receptor agonist type, dosing regimen, exposure definition, and therapy duration), surgical characteristics (fusion region and surgery type), and outcome variables. Key outcome measures extracted for this meta-analysis included postoperative complications, namely, acute kidney injury, cardiovascular accidents, DVT, myocardial infarction, pneumonia, pseudoarthrosis, infection, surgical transfusion requirements, readmission rates, and reoperation rates. For every outcome, quantitative data were extracted for the GLP-1 receptor agonist and control groups, including event numbers, total sample sizes, percentages, and confidence intervals (CIs) where available.

Risk of Bias Assessment

The risk of bias for every study in the review was assessed by two independent reviewers using the Newcastle-Ottawa Scale (NOS), which is specifically designed for evaluating observational studies [16]. The NOS evaluates study quality across the following three domains: selection of study groups, comparability of groups, and ascertainment of exposure or outcome. Each study was systematically assessed for potential sources of bias, including participant selection criteria, adequacy of follow-up, outcome measurement methods, and control for confounding variables. Any disagreements between reviewers regarding bias assessment were resolved through collaborative discussion or consultation with a third reviewer when consensus could not be reached. The overall risk of bias for each study was categorized as poor, fair, or good based on the NOS scoring system.

Statistical Analysis

Statistical analysis was performed using Review Manager (RevMan) version 5.4 software, with statistical significance set at a p-value <0.05. For dichotomous outcomes, risk ratios (RRs) with 95% CIs were calculated, while mean differences (MDs) with 95% CIs were computed for continuous variables. Heterogeneity between studies was evaluated using the I² statistic and chi-square test, with data considered heterogeneous when the chi-square test produced a p-value <0.1 or when I² values exceeded 50%. A fixed-effects model was employed for homogeneous data, whereas a random-effects model was applied when significant heterogeneity was detected between studies.

Results

Study Selection

**Figure 1:**
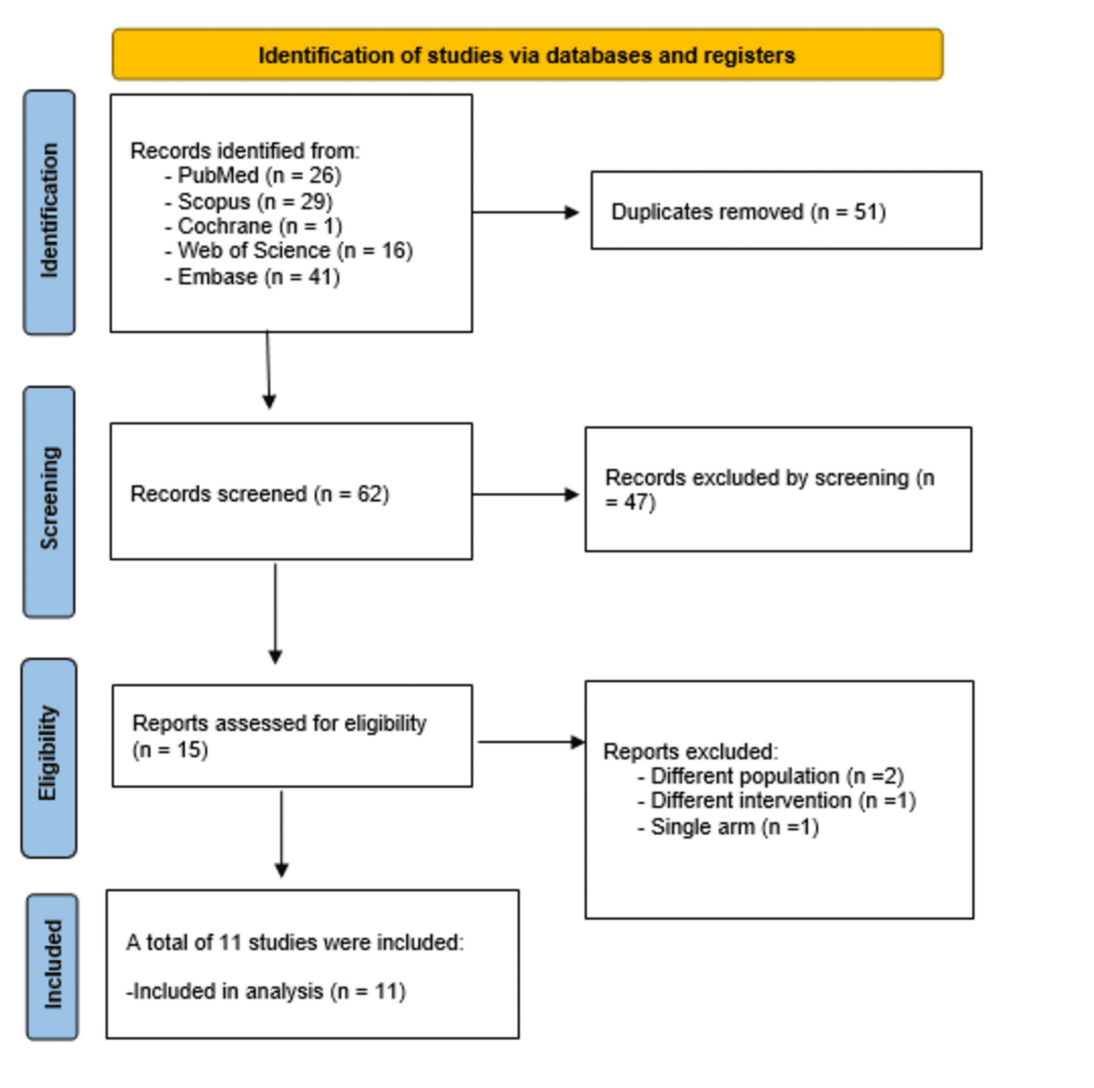
Preferred Reporting Items for Systematic Reviews and Meta-Analyses (PRISMA) flow diagram.

After searching four databases, we collected 113 records. Removing 51 duplicates led to 62 unique records. We then screened the titles and abstracts, excluding 47 entries. We retrieved the full text of the remaining 15 records and evaluated them against our eligibility criteria. During this evaluation, we excluded four irrelevant studies. Finally, 11 studies were included in the systematic review and meta-analysis [17-27]. The flow diagram illustrating the study selection is shown in Figure 1.

Characteristics of the Included Studies

This systematic review included 11 retrospective observational studies comprising a total of 90,805 participants, with final matched cohorts totalling 14,344 participants across all studies. All studies were conducted in the United States except for one global multi-center study (Chang et al., 2025) [18]. All included studies were published in the last two years. The mean age of participants ranged from 55 to 64 years across studies, with a relatively balanced gender distribution, ranging in male gender from 32.1% to 55.9%. Body mass index (BMI) varied considerably, from 26.2 to 35.1 kg/m², implying that the included population contained normal BMI and obese participants. The prevalence of diabetes mellitus ranged from 0% in non-diabetic cohorts to 100% in diabetes-specific studies. Hypertension was highly prevalent across studies, affecting 42.4% to 94.6% of participants. Obesity or overweight status was present in 52% to 100% of patients. Various surgical procedures were used. Most studies defined GLP-1 receptor agonist exposure as prescription within six months before or after surgery, with some studies extending the window to one year before surgery. Follow-up periods ranged from 90 days to 3 years, with most studies providing intermediate-term follow-up at 1-2 years. Full details of baseline characteristics and the summary of the included studies are shown in Table 1 and Table 2.

**Table 1:** Baseline characteristics of the included studies. ACDF = anterior cervical discectomy and fusion; AKI = acute kidney injury; CSDF = cervical spine decompression and fusion; ED = emergency department; GLP-1 = glucagon-like peptide-1; PCF = posterior cervical fusion; PLIF = posterior lumbar interbody fusion; PLF = posterior lumbar fusion; RA = receptor agonist; T2DM = type 2 diabetes mellitus; TLIF = transforaminal lumbar interbody fusion; UTI = urinary tract infection

Study ID	Age (years, mean ± SD)		Gender (male, %)		BMI (kg/m², mean ± SD)		Diabetes mellitus (%)		Hypertension (%)		Obesity/Overweight (%)		Smoking (%)	
	GLP-1	Control	GLP-1	Control	GLP-1	Control	GLP-1	Control	GLP-1	Control	GLP-1	Control	GLP-1	Control
Agrawal et al. (2025) [17]	61.0 ± 10.3	61.3 ± 12.1	47.1	46.7	35.1 ± 6.6	29.9 ± 6.2	79.10%	79.40%	76.3	76.7	52.3	52	21	22.4
Chang et al. (2025) [18]	58.6 ± 10.4	59.1 ± 11.1	47.2	46.7	NA	NA	100	100	82	82.2	63.3	64.9	20.1	18.2
Chang et al. (2025) [27]	62.8 ± 9.9	63.2 ± 10.3	40	40	NA	NA	100	100	88.2	88.6	64.6	64.3	19.7	13.8
Ghali et al. (2025) [26]	61.7 ± 10.4	61.8 ± 11.0	42.5	42.5	NA	NA	78	78.3	78.7	79.5	NA	NA	NA	NA
Goldman et al. (2025) [19]	61.4 ± 9.1	62.0 ± 12.1	37.9	37.5	33.8	33.5	100	100	58.5	60.1	NA	NA	NA	NA
Khalid et al. (2025) [20]	55-59 (most common range)	55-59 (most common range)	32.1	32.1	more than 35	more than 35	0	0	79	79	72	72	32.3	32.3
Ng et al. (2025) [21]	60.44 ± 9.65	60.47 ± 10.31	43.2	44	NA	NA	100	100	42.4	40.8	77.1	58.3	46.7	49.1
Seddio et al. 2025 [22]	60.63 ± 8.45	60.61 ± 8.18	40.3	40.1	NA	NA	100	100	94.6	89.3	77.5	77.3	47.6	47.6
Tao et al. 2025 [23]	Range from 60 to 64 for the majority	Range from 60 to 64 for the majority	53.4	52.1	NA	NA	100	100	94.6	89	70.1	36.6	38.6	33.9
Tummala et al. (2025) [24]	60.8 ± 11.0	61.0 ± 11.1	45.4	45.5	26.5 ± 4.8	26.2 ± 3.9	67.6	67.7	67.8	67.6	100 in the obese cohort and 0 in the non-obese	100 in the obese cohort and 0 in the non-obese	4.8	3.9
Wiener et al. (2025) [25]	62.2 ± 9.7	62.3 ± 11.3	42.1	55.9	26.5 ± 4.8	26.2 ± 3.9	100	100	82.5	52	100 in the obese cohort and 0 in the non-obese	100 in the obese cohort and 0 in the non-obese	26.7	18.8

**Table 2:** Summary of the included studies. BMI = body mass index (measured in kg/m²); GLP-1 = glucagon-like peptide-1; SD = standard deviation; NA = not available; PLF = posterior lumbar fusion; PCF = posterior cervical fusion

Study ID	Study design	Total patients	Country	Intervention	Surgery type	GLP-1 exposure definition	Fusion Region	Outcome	Follow-up	Time points	Conclusion
Agrawal et al. (2025) [17]	Retrospective cohort study	37,147 (709 matched pairs)	Global (predominantly US)	GLP-1 agonist use vs. non-use	Single-level lumbar fusion (PLIF/TLIF)	Any GLP-1 agonist within 6 months before or after surgery	Lumbar	Pseudarthrosis	2 years	6 months, 1 year, 2 years	In this cohort study, patients who were prescribed GLP-1 agonists in the perioperative period had reduced rates of pseudarthrosis compared with patients without GLP-1 agonist prescriptions. These findings suggest a potential therapeutic benefit of GLP-1 agonists in enhancing spinal fusion outcomes and warrant further prospective studies to confirm these results and explore the underlying mechanisms
Chang et al. (2025) [18]	Retrospective cohort study	14,764 (1,242 matched pairs)	Global (47% US, Europe, South America, Asia)	GLP-1 agonist use vs. non-use	Single-level anterior cervical discectomy and fusion (ACDF)	Any GLP-1 agonist within 6 months before or after surgery	Lumbar (posterior)	Pseudarthrosis	2 years	6 months, 1 year, 2 years	Perioperative GLP-1 drug use is associated with a lower risk of pseudarthrosis in T2DM patients undergoing ACDF. These findings suggest a potential role for GLP-1 drugs in improving spinal fusion outcomes
Chang et al. (2025) [27]	Retrospective cohort study	16,973 (884 matched pairs)	Global (117 healthcare systems)	Semaglutide use vs. non-use	Posterior lumbar fusion	Preoperative semaglutide exposure	Cervical (ACDF)	Pseudarthrosis	2 years	6 months, 1 year, 2 years	Semaglutide use is associated with a reduced risk of pseudarthrosis following posterior lumbar fusion in patients with T2DM. Further studies are warranted to elucidate the mechanisms underlying this potential benefit and assess its implications in broader patient populations
Ghali et al. (2025) [26]	Retrospective cohort study	2,180 (1,090 matched pairs)	USA	GLP-1 RA use vs. non-use	Lumbar spine procedures	GLP-1 RA within 6 months preoperatively	Lumbar	Pseudarthrosis, 90-day complications	1 year	90 days, 1 year	The effects of GLP-1 receptor agonists on patients undergoing lumbar spine surgery do not increase the risk of adverse outcomes and should not be a reason to exclude patients from undergoing lumbar spinal procedures. Risk of medical and mechanical complications is comparable to that of control patients when statistically controlled for other comorbidities
Goldman et al. (2025) [19]	Retrospective propensity-matched cohort study	1,385 (277 GLP-1 RA, 1,108 controls)	USA	Preoperative GLP-1 RA use vs. non-use	Spinal decompression and/or fusion	GLP-1 RA prescribed preoperatively	Mixed (lumbar, cervical)	Length of stay, readmission	90 days	90 days, 1 year	Preoperative GLP-1 RA use was associated with a statistically significant reduction in postoperative length of stay among patients undergoing spinal decompression and/or fusion, particularly among patients undergoing lumbar fusion
Khalid et al. (2025) [20]	Retrospective matched cohort study	942 (471 matched pairs)	USA	Semaglutide use vs. non-use	One- to three-level transforaminal lumbar interbody fusion (TLIF)	Semaglutide prescription for weight loss	Lumbar (TLIF)	Reoperation; pneumonia, UTI, AKI	Mixed	30 days, 12 months	Semaglutide may adversely affect lumbar fusion outcomes and necessitate additional surgery, possibly secondary to its systemic effects on bone metabolism and weight loss patterns. Further research into optimal drug formulation, dosage, and weight loss protocols will be required before mainstream use
Ng et al. (2025) [21]	Retrospective cohort	1,880 (340 semaglutide, 1,540 controls)	USA	Semaglutide vs. no semaglutide	Posterior cervical fusion (PCF)	Active semaglutide prescription before surgery	Cervical (PCF)	Pseudarthrosis, Dysphagia, and cost	2 years	90 days and 2 years	Semaglutide use is associated with an increased risk of long-term complications, including pseudoarthrosis and dysphagia, as well as lower same-day and 90-day costs in patients undergoing PCF. These findings highlight the importance of careful perioperative management of semaglutide users to optimize outcomes while leveraging its purported benefits
Seddio et al. 2025 [22]	Retrospective cohort	1,476 (339 semaglutide, 1,137 controls)	USA	Semaglutide vs. no semaglutide	Single-level posterior lumbar fusion (PLF)	Semaglutide use within 1 year before surgery	Lumbar (posterior)	Adverse events, ED visits	90 days	90 days	The current study found consistent reductions in aggregated 90-day adverse events, but similar odds of hospital readmission for T2DM patients undergoing PLF taking semaglutide preoperatively. These encouraging findings of reduced postoperative complications suggest further prospective analysis, as the observed findings suggest clinical benefit to semaglutide being utilized by the studied patient population
Tao et al. 2025 [23]	Retrospective cohort	596 (298 semaglutide, 298 controls)	USA	Semaglutide vs. no semaglutide	Cervical spine decompression and fusion (CSDF)	Semaglutide treatment within 6 months before surgery	Cervical (decompression + fusion)	Surgical complications (short-term)	<6 months	6 months	This study suggests that in patients with T2DM, semaglutide treatment is not associated with higher rates of short-term adverse events after CSDF. The effect of semaglutide use on long-term outcomes remains unknown
Tummala et al. (2025) [24]	Retrospective cohort study	5,722 (2,861 matched pairs)	USA	GLP-1 RA use vs. non-use	Elective lumbar spine surgery (arthrodesis, facetectomy, foraminotomy, laminectomy, laminotomy, disc/joint excision)	GLP-1 RA prescription within 6 months before surgery	Lumbar (elective)	Pseudarthrosis	3 years	90 days, 1 year, 3 years	Preoperative GLP-1 RA use was not associated with increased short- or intermediate-term medical or mechanical complications following lumbar spine procedures. Notably, GLP-1 RA use correlated with reduced rates of pseudarthrosis at 1- and 3-year intervals
Wiener et al. (2025) [25]	Retrospective cohort study	15,000+ (2,263 final cohort: 1,560 obese, 703 non-obese)	USA	GLP-1 RA use vs. non-use	Spinal fusion (diabetic patients on metformin)	GLP-1 RA prescription with at least 1 year follow-up	Spine (mixed)	Infections, Revisions, Readmissions, and Mobility Impairments	Mid-term?	90 days, 1 year	GLP-1 RA use in spinal fusion patients was associated with improved postoperative outcomes, including lower infection rates, fewer revisions, and better quality of life metrics. These findings suggest that GLP-1 RAs may be a valuable adjunctive therapy in managing surgical outcomes in diabetic and obese patients undergoing spinal fusion. Further prospective and animal-based studies are needed to confirm these findings and explore the underlying mechanisms

Quality Assessment

The methodological quality of the included studies was assessed using the NOS tool. Ten studies demonstrated good quality, while one study (Khalid et al., 2025) was rated as fair quality (Table 3).

**Table 3:** Newcastle-Ottawa Scale assessment. References: [17-27]. Each star (*) represents fulfillment of a specific quality criterion according to the Newcastle-Ottawa Scale.

ID	Newcastle-Ottawa Scale assessment								
	Selection				Comparability	Outcome			Quality Score
	Representativeness of the exposed cohort	Selection of the non-exposed cohort	Ascertainment of exposure	Demonstration that the outcome of interest was not present at the start of the study	Comparability of cohorts based on the design or analysis	Assessment of outcome	Was the follow-up long enough for outcomes to occur	Adequacy of follow-up of cohorts	
Agrawal et al. (2024) [17]	*	*	*	*	**	*	*	*	good
Chang et al. (2025) [27]	*	*	*	*	**	*	*	*	good
Chang et al. (2025) [18]	*	*	*	*	**	*	*	*	good
Ghali et al. (2025) [26]	*	*	*		*	*	*	*	good
Goldman et al. (2025) [19]	*	*	*	*	**	*	*	*	good
Khalid et al. (2025) [20]		*	*		**	*	*	*	fair
Ng et al. (2025) [21]	*	*	*		*	*	*	*	good
Seddio et al. (2025) [22]	*	*	*	*	*	*	*	*	good
Tao et al. (2025) [23]	*	*	*	*	*	*	*	*	good
Tummala et al. (2025) [24]	*	*	*		**	*	*	*	good
Wiener et al. (2025) [25]	*	*	*	*	**	*	*	*	good

Primary Outcomes

**Figure 2:**
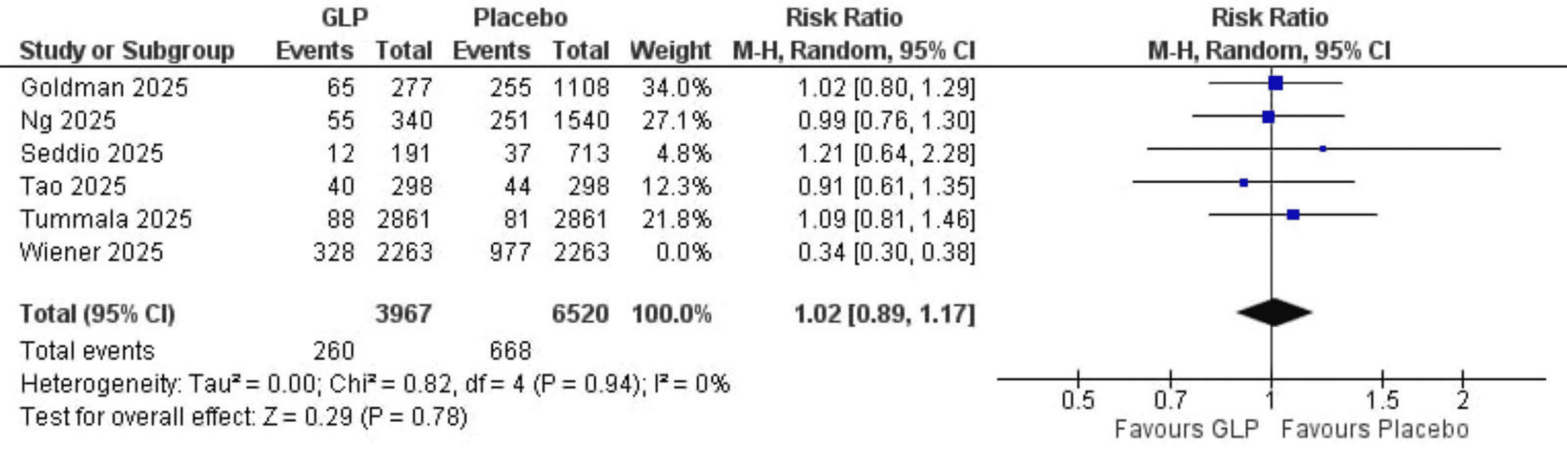
Readmission rate. References: [19,21,22,23,24,25]. GLP-1 = glucagon-like peptide-1; CI = confidence interval

Readmission rate: Six studies were included in the analysis, with a total of 15,013 participants. Despite the results favoring the GLP-1 group, the difference was not statistically significant (RR = 0.85, 95% CI = 0.48-1.51; p = 0.58). The results are heterogeneous (I² = 97%). We could solve heterogeneity by excluding Wiener et al. (2025) [25] by the leave-one-out method (RR = 1.02, 95% CI = 0.89-1.17; p = 0.78) (Figure 2).

**Figure 3:**
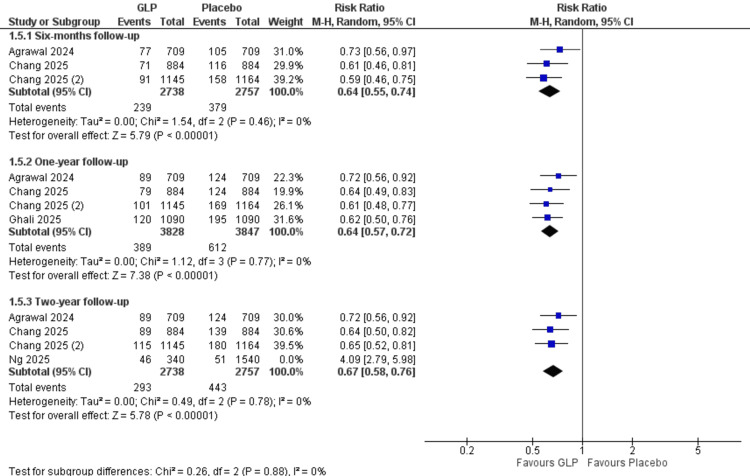
Pseudarthrosis at different follow-up periods. References: [17,18,26,27]. GLP-1 = glucagon-like peptide-1; CI = confidence interval

Pseudoarthrosis: At the six-month follow-up, by analyzing three studies with 5,495 participants, GLP-1 receptor agonists demonstrated a significant protective effect compared to control (RR = 0.63, 95% CI = 0.54-0.74; p < 0.00001). At the one-year follow-up, by analyzing four studies with 7,675 participants, GLP-1 receptor agonists demonstrated a significant protective effect compared to control (RR = 0.64, 95% CI = 0.57-0.72; p < 0.00001). At two-year follow-up, by analyzing four studies with 7,375 participants, GLP-1 receptor agonists demonstrated no significant difference in effect compared to control (RR = 1.03, 95% CI = 0.53-2.03; p = 0.92). The heterogeneity was solved by using the leave-one-out method (I² = 0%) (Figure 3).

**Figure 4:**
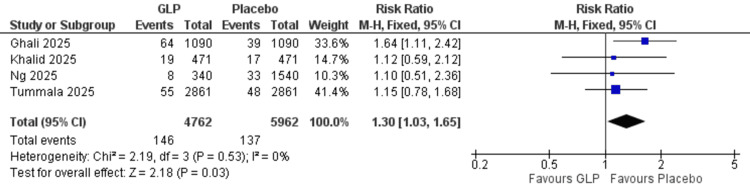
Acute kidney injury. References: [20,21,24,26]. GLP-1 = glucagon-like peptide-1; CI = confidence interval

Acute kidney injury: Four studies (Ghali et al. (2025), Khalid et al. (2025), Ng et al. (2025), Tummala et al. (2025)) [20,21,24,26] with 10,724 participants demonstrated an increased incidence of acute kidney injury compared to the GLP-1 group (RR = 1.30, 95% CI = 1.03-1.65, p = 0.03). The analysis showed homogeneity (I² = 0%) (Figure 4).

**Figure 5:**
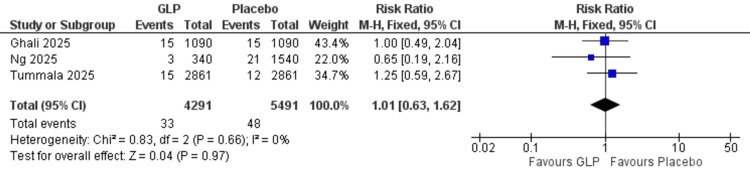
Cerebrovascular accidents. References: [21,24,26]. GLP-1 = glucagon-like peptide-1; CI = confidence interval

Cerebrovascular accident: No significant difference was observed between GLP-1 receptor agonists and placebo for this endpoint (RR = 1.01, 95% CI = 0.63-1.62; p = 0.97; I² = 0%). The analysis showed homogeneity (I² = 0%) (Figure 5).

**Figure 6:**
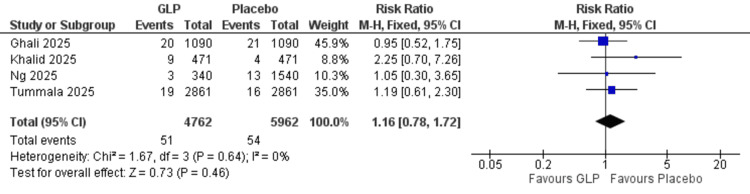
Deep vein thrombosis. References: [20,21,24,26]. GLP-1 = glucagon-like peptide-1; CI = confidence interval

DVT incidence: No significant difference was observed between GLP-1 receptor agonists and placebo for the DVT incidence (RR = 1.16, 95% CI = 0.78-1.72; p = 0.46; I² = 0%) (Figure 6).

**Figure 7:**
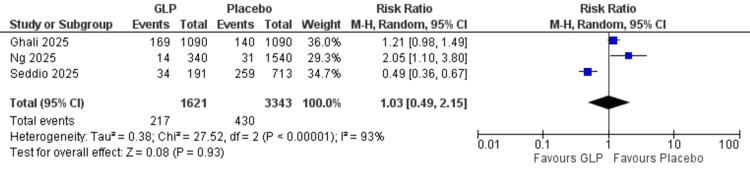
Emergency department visit. References: [21,22,26]. GLP-1 = glucagon-like peptide-1; CI = confidence interval

Emergency department visit: No significant difference was observed between GLP-1 receptor agonists and emergency department visit (RR = 1.03, 95% CI = 0.49-2.15; p = 0.93). The analysis showed heterogeneity (I² = 93%) (Figure 7).

**Figure 8:**
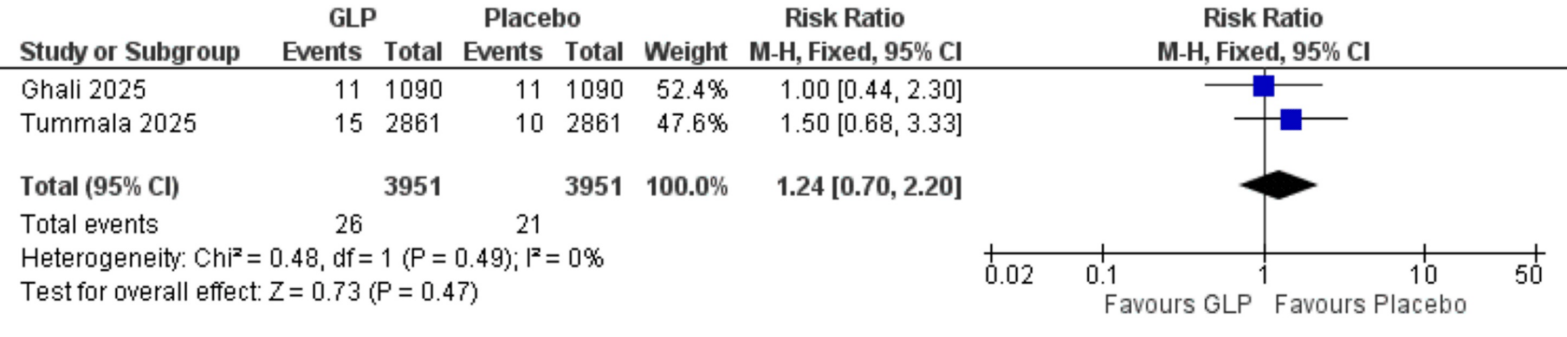
Myocardial infarction. References: [24,26]. GLP-1 = glucagon-like peptide-1; CI = confidence interval

Myocardial infarction: This analysis was limited to two studies (Ghali et al. (2025) and Tummala et al. (2025)) [24,26] with 7,902 participants. No significant difference was found between groups (RR = 1.24, 95% CI = 0.70-2.20; p = 0.47), and the data were homogeneous (I² = 0%) (Figure 8).

**Figure 9:**
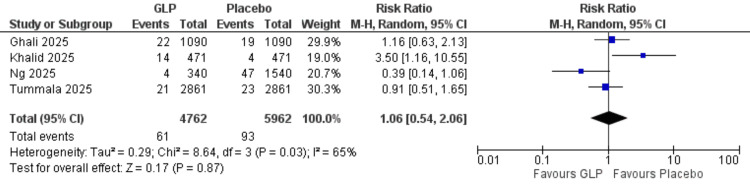
Pneumonia incidence. References: [20,21,24,26]. GLP-1 = glucagon-like peptide-1; CI = confidence interval

Pneumonia incidence: No significant difference was observed between the GLP-1 group and comparator in four of the analyzed studies (RR = 1.06, 95% CI = 0.54-2.06; p = 0.87). The data were heterogeneous, and heterogeneity could not be resolved with traditional methods (I² = 65%) (Figure 9).

**Figure 10:**
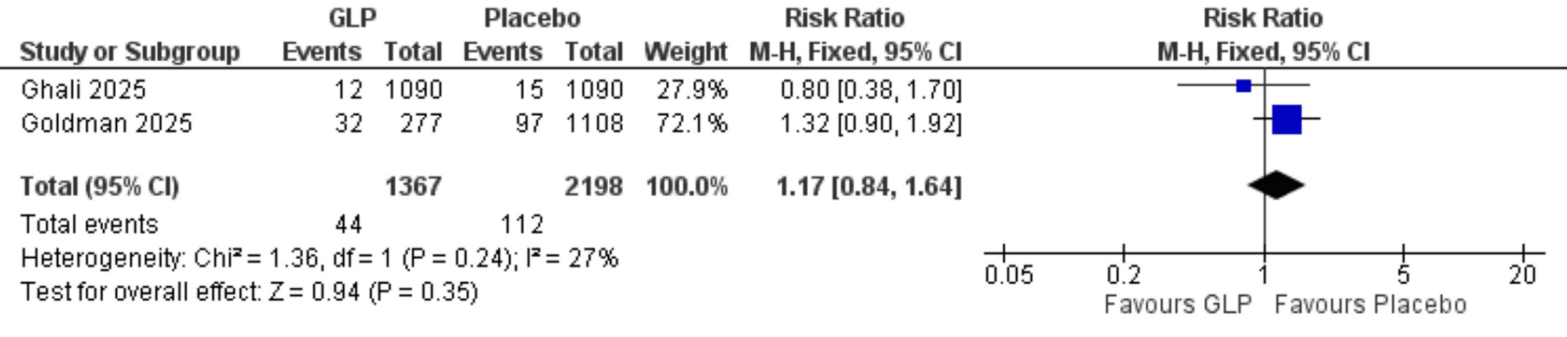
Reoperation rate. References: [19,26]. GLP-1 = glucagon-like peptide-1; CI = confidence interval

Reoperation rate: This analysis was limited to two studies with 44 events in 1,367 GLP participants and a total of 3,565 participants [19,26]. No significant difference was observed (RR = 1.17, 95% CI = 0.84-1.64; p = 0.35; I² = 27%) (Figure 10).

**Figure 11:**
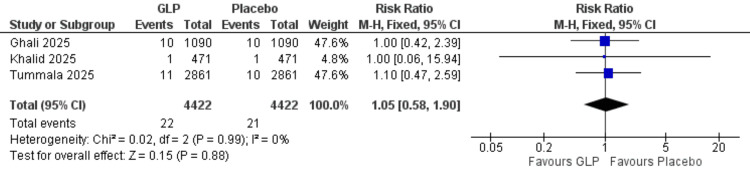
Pulmonary embolism. References: [20,24,26]. GLP-1 = glucagon-like peptide-1; CI = confidence interval

Pulmonary embolism: In the analysis of three studies containing 8,844 patients [20,24,26], GLP-1 receptor agonists showed no significant effect compared to placebo (RR = 1.05, 95% CI = 0.58-1.90; p = 0.88), and the data were homogeneous (I² = 0%) (Figure 11).

**Figure 12:**
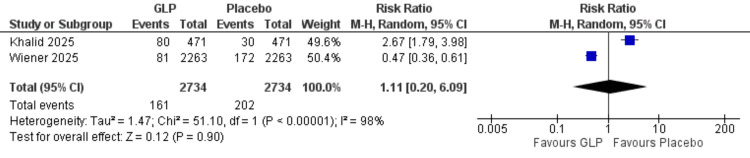
Revision surgery. References: [20,25]. GLP-1 = glucagon-like peptide-1; CI = confidence interval

Revision surgery: No significant difference was found (RR = 1.11, 95% CI = 0.20-6.09; p = 0.90), and substantial heterogeneity was present (I² = 98%) (Figure 12).

**Figure 13:**
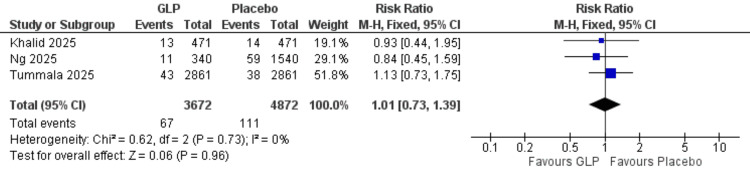
Surgical site infection. References: [20,21,24]. GLP-1 = glucagon-like peptide-1; CI = confidence interval

Surgical site infection: GLP-1 receptor agonists showed no significant effect regarding infection rate within the site (RR = 1.01, 95% CI = 0.73-1.39; p = 0.96; I² = 0%) (Figure 13).

Subgroup Analysis Results

Acute Kidney Injury (Patient Population Subgroup)

Mixed population: Two studies (Ghali et al. (2025), Tummala et al. (2025)) [24,26] with 7,902 participants demonstrated a significant increase in acute kidney injury incidence with GLP-1 receptor agonists (RR = 1.37, 95% CI = 1.04-1.80; p = 0.02). Low heterogeneity was observed (I² = 40%).

Diabetic population: One study (Ng et al., 2025) [21] with 1,880 participants showed no significant difference between groups (RR = 1.10, 95% CI = 0.51-2.36]; p = 0.81).

**Figure 14:**
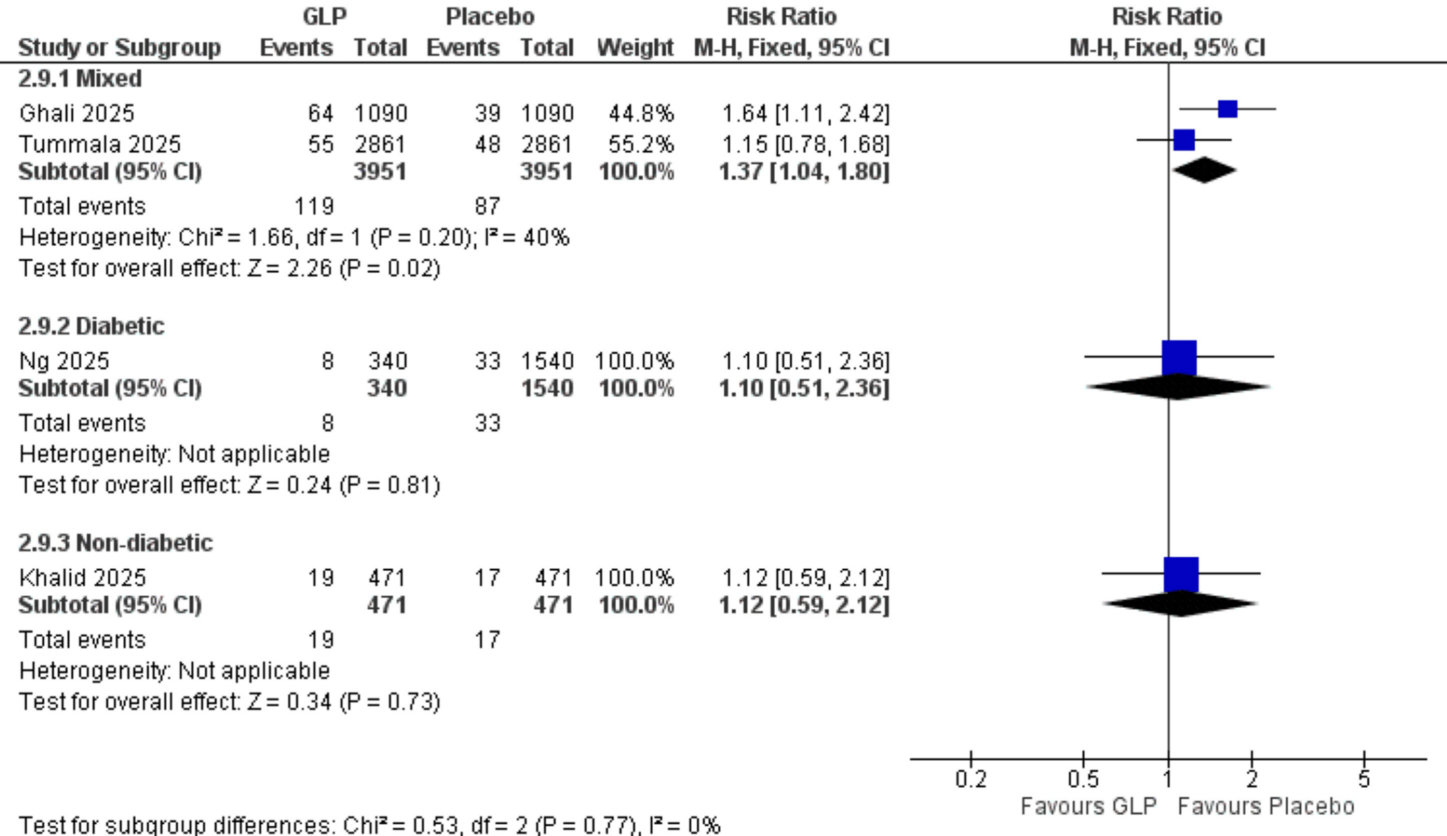
Acute kidney injury (patient population subgroup). References: [20,21,24,26]. GLP-1 = glucagon-like peptide-1; CI = confidence interval

Non-diabetic population: One study (Khalid et al., 2025) [20] with 942 participants demonstrated no significant difference (RR = 1.12, 95% CI = 0.59-2.12; p = 0.73). All data are shown in Figure 14.

Acute Kidney Injury (Surgical Procedure Subgroups)

Lumbar fusion: Three studies (Ghali et al. (2025), Khalid et al. (2025), Tummala et al. (2025)) [20,24,26] with 8,844 participants showed a significant increase in acute kidney injury with GLP-1 receptor agonists (RR = 1.33, 95% CI = 1.03-1.70; p = 0.03). No heterogeneity was observed (I² = 0%).

**Figure 15:**
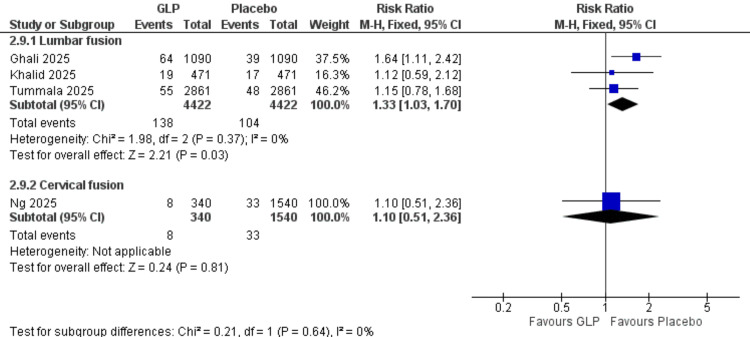
Acute kidney injury (surgery subgroup). References: [20,21,24,26]. GLP-1 = glucagon-like peptide-1; CI = confidence interval

Cervical fusion: One study (Ng et al., 2025) [21] with 1,880 participants demonstrated no significant difference (RR = 1.10, 95% CI = 0.51-2.36; p = 0.81). All data are shown in Figure 15.

DVT Incidence (Patient Population Subgroups)

Mixed population: Two studies (Ghali et al. (2025), Tummala et al. (2025)) [24,26] with 7,902 participants showed no significant difference (RR = 1.05, 95% CI = 0.67-1.65; p = 0.82). No heterogeneity was observed (I² = 0%).

Diabetic population: One study (Ng et al., 2025) [21] with 1,880 participants demonstrated no significant difference (RR = 1.05, 95% CI = 0.30-3.65; p = 0.94).

**Figure 16:**
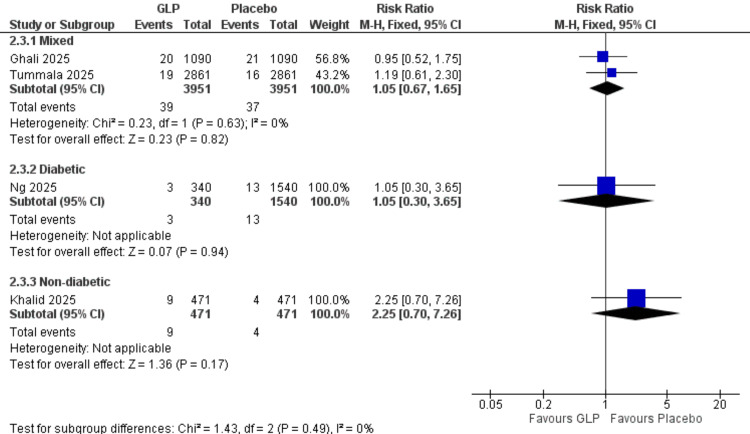
DVT subgroup by diabetic state. Reference: [20,21,24,26]. GLP-1 = glucagon-like peptide-1; CI = confidence interval

Non-diabetic population: One study (Khalid et al., 2025) [20] with 942 participants showed no significant difference (RR = 2.25, 95% CI = 0.70-7.26; p = 0.17). All data are shown in Figure 16.

Pneumonia Incidence (Patient Population Subgroups)

Mixed population: Two studies (Ghali et al. (2025), Tummala et al. (2025)) [24,26] with 7,902 participants demonstrated no significant difference (RR = 1.02, 95% CI = 0.67-1.56; p = 0.91). No heterogeneity was observed (I² = 0%).

Diabetic population: One study (Ng et al., 2025) [21] with 1,880 participants showed no significant difference (RR = 0.39, 95% CI = 0.14-1.06; p = 0.07).

**Figure 17:**
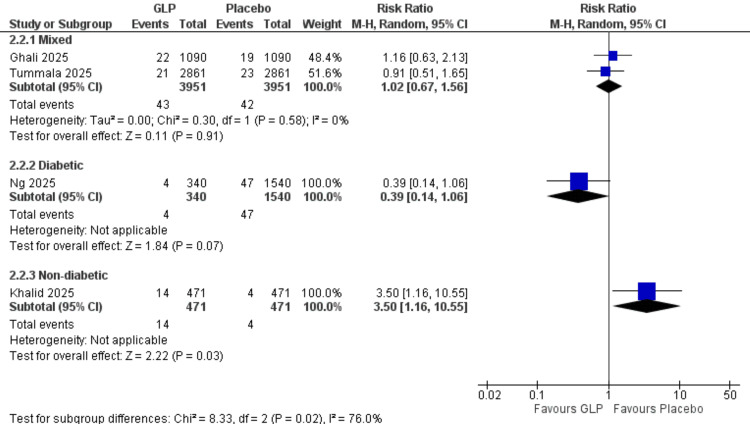
Pneumonia patient subgroups. References: [20,21,24,26]. GLP-1 = glucagon-like peptide-1; CI = confidence interval

Non-diabetic population: One study (Khalid et al., 2025) [20] with 942 participants demonstrated a significant increase in pneumonia incidence (RR = 3.50, 95% CI = 1.16-10.55; p = 0.03). All data are shown in Figure 17.

Pneumonia Incidence (Surgical Procedure Subgroups)

Lumbar fusion: Three studies (Ghali et al. (2025), Khalid et al. (2025), Tummala et al. (2025)) [20,24,26] with 8,844 participants showed no significant difference (RR = 1.33, 95% CI = 0.71-2.49; p = 0.37). High heterogeneity was present (I² = 55%).

**Figure 18:**
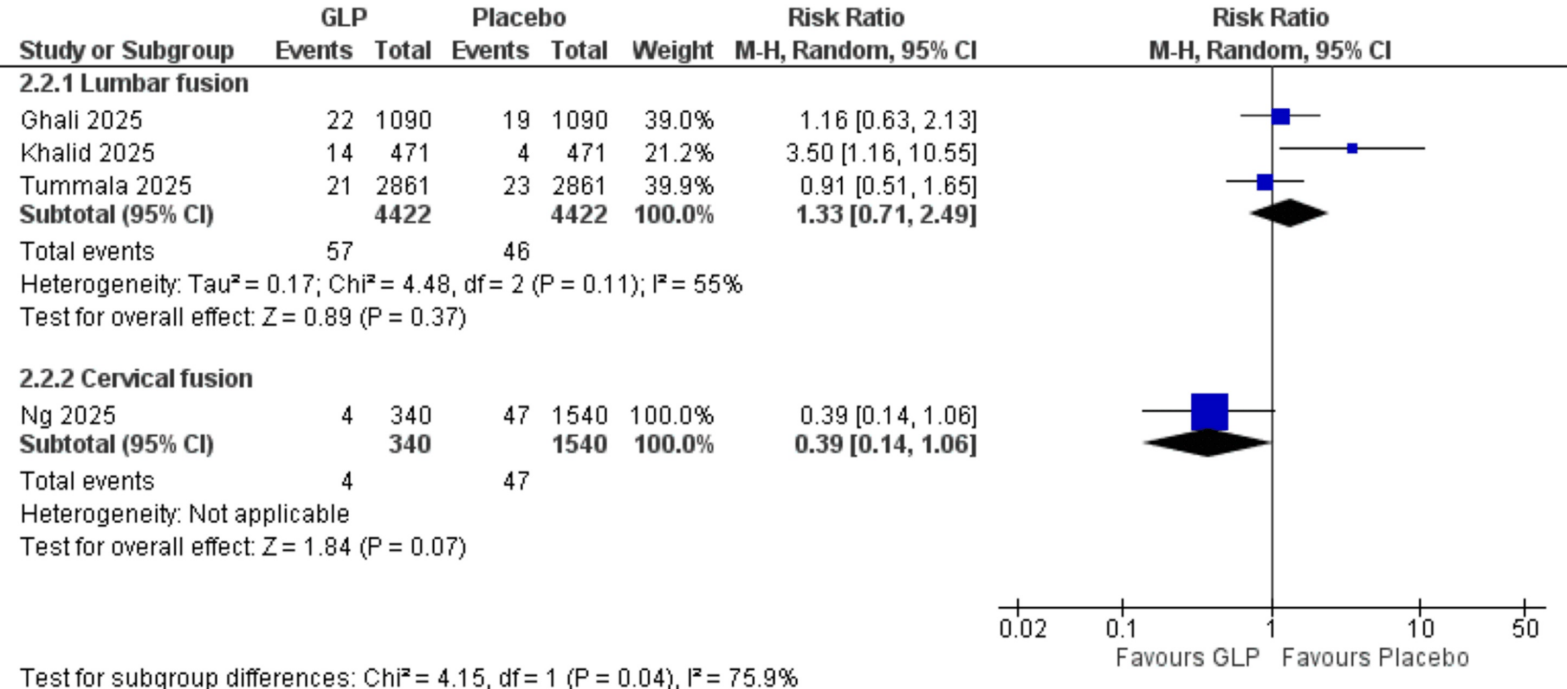
Pneumonia (surgery subgroups). References: [20,24,26]. GLP-1 = glucagon-like peptide-1; CI = confidence interval

Cervical fusion: One study (Ng et al., 2025) [21] with 1,880 participants demonstrated no significant difference (RR = 0.39, 95% CI = 0.14-1.06; p = 0.07). All data are shown in Figure 18.

Pseudoarthrosis at 24 Months (Patient Population Subgroups)

Mixed population: One study (Agrawal et al., 2024) [17] with 1,418 participants showed a significant protective effect (RR = 0.72, 95% CI = 0.56-0.92; p = 0.010).

**Figure 19:**
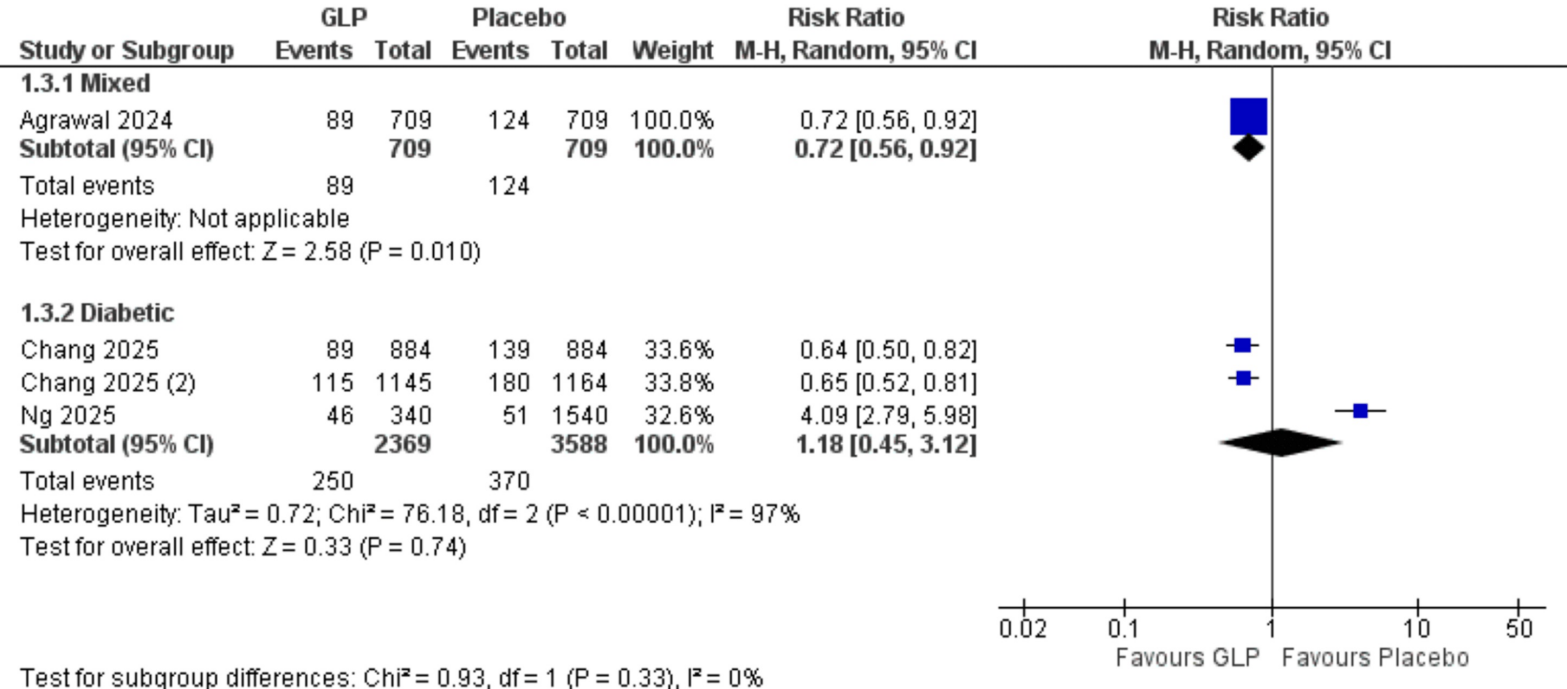
Pseudarthrosis patient subgroups. References: [17,18,21,27]. GLP-1 = glucagon-like peptide-1; CI = confidence interval

Diabetic population: Three studies with 5,957 participants demonstrated no significant difference (RR = 1.18, 95% CI = 0.45-3.12; p = 0.74). High heterogeneity was observed (I² = 97%). All data are shown in Figure 19.

Pseudoarthrosis at 24 Months (Surgical Procedure Subgroups)

Cervical fusion: Two studies with 4,189 participants showed no significant difference (RR = 1.62, 95% CI = 0.27-9.85; p = 0.60). Very high heterogeneity was present (I² = 99%).

**Figure 20:**
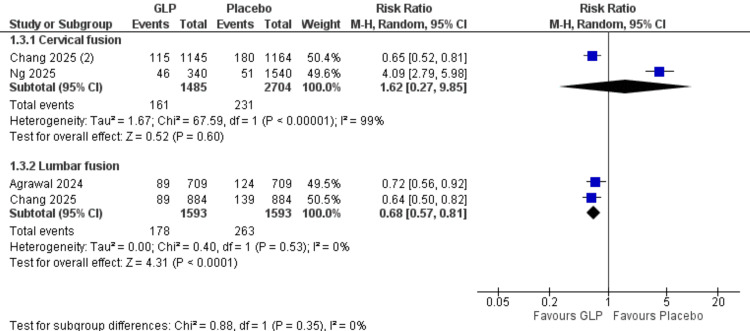
Pseudarthrosis (surgical subgroups). References: [17,18,21,27]. GLP-1 = glucagon-like peptide-1; CI = confidence interval

Lumbar fusion: Two studies with 3,277 participants demonstrated a significant protective effect (RR = 0.68, 95% CI = 0.57-0.81; p < 0.0001). No heterogeneity was observed (I² = 0%). All data are shown in Figure 20.

Readmission Rate (Surgical Procedure Subgroups)

Unspecified: Two studies (Goldman et al. (2025), Wiener et al. (2025)) [19,25] with 5,911 participants showed a significant protective effect (RR = 0.58, 95% CI = 0.20-1.73; p = 0.33). Very high heterogeneity was present (I² = 99%).

Lumbar fusion: Two studies (Seddio et al. (2025), Tummala et al. (2025)) [22,24] demonstrated no significant difference (RR = 1.11, 95% CI = 0.85-1.45; p = 0.45). No heterogeneity was observed (I² = 0%).

**Figure 21:**
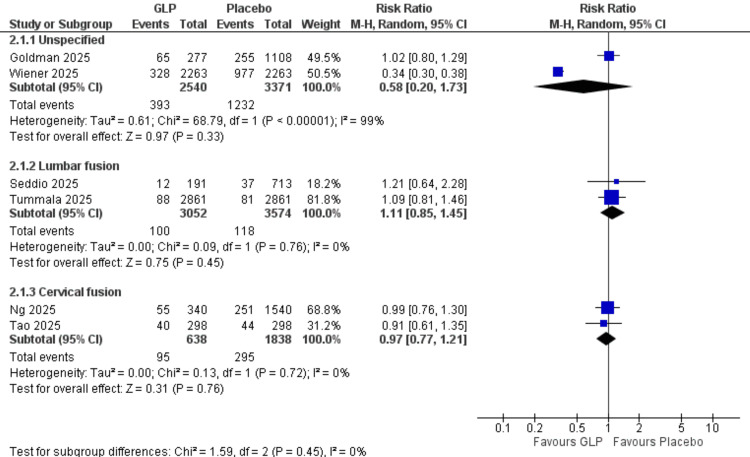
Readmission rate surgery subgroups. References: [19,21-25]. GLP-1 = glucagon-like peptide-1; CI = confidence interval

Cervical fusion: Two studies (Ng et al. (2025), Tao et al. (2025)) [21,23] showed a significant protective effect (RR = 0.97, 95% CI = 0.77-1.21; p = 0.76). No heterogeneity was observed (I² = 0%). All data are shown in Figure 21.

Discussion

This meta-analysis of 14,344 participants demonstrated that GLP-1 receptor agonists significantly reduced pseudoarthrosis rates at the 6-month and 12-month follow-ups. On the other hand, the drug showed increased acute kidney injury risk and no sustained benefit in pseudoarthrosis at 24 months. Neutral effects were observed for other complications. Subgroup analysis revealed that the protective effects were particularly pronounced in lumbar fusion procedures compared to cervical procedures and in mixed populations compared to diabetes-specific cohorts.

All included studies were conducted and published in the last two years, as the topic is a very recent and rapidly emerging area of research interest. Moreover, the clustering of publications suggests that there was a recognized knowledge gap in understanding the perioperative effects of GLP-1 receptor agonists in spinal surgery [28]. Different databases have shown conflicting results regarding GLP-1 receptor agonists in spinal fusion, with a previous study based on the PearlDiver database showing no benefits [23]. In contrast, another study based on TriNetX demonstrated positive effects, highlighting the need for a meta-analysis to resolve these controversies [25]. The rising spinal fusion rates underscore the need to understand how patient conditions and medications affect surgical outcomes to reduce complications and improve care [10].

Despite the well-established renal protective effects of GLP-1 receptor agonists in cardiovascular and diabetes outcome trials, our analysis revealed an unexpected increase in acute kidney injury risk, suggesting that perioperative physiological stresses may alter the typical nephroprotective profile of these agents. This can result from several mechanisms, including perioperative volume depletion from GLP-1-induced nausea and vomiting, drug-drug interactions with nephrotoxic perioperative medications, altered hemodynamics from combined anesthetic and GLP-1 effects, and prolonged dehydration secondary to delayed gastric emptying [29-31].

GLP-1 receptor agonists were initially developed and approved for glycemic control in patients with type 2 diabetes mellitus and subsequently demonstrated efficacy in weight reduction [32] and cardiovascular risk reduction. Some spinal fusion patients may have benefited from the drug when used for other conditions. Despite comorbidities worsening surgical outcomes, these multimorbid patients may still derive clinical benefit in the orthopedic surgical setting [33,34].

Due to the mechanism of GLP-1 agonists, as they bind to GLP-1 receptors, and upon activation, these receptors trigger multiple downstream effects, including enhanced insulin production, prevention of pancreatic beta-cell apoptosis, and suppression of glucagon release [35]. These glycemic control mechanisms are particularly relevant for spinal fusion outcomes, as elevated HbA1c levels and diabetes mellitus have been independently associated with impaired bone healing, increased infection rates, and higher rates of pseudarthrosis in spinal fusion procedures [33]. Obese patients also benefit from the mechanism, as the drug induces delayed gastric emptying and increased satiety through their action on receptors in the enteric nervous system [36]. These mechanisms that lead to weight loss are clinically significant in spinal fusion contexts, as elevated BMI has been consistently linked to adverse surgical outcomes, including prolonged operative times, increased perioperative complications, delayed wound healing, and higher rates of hardware failure [34]. Additionally, recent research shows GLP-1 receptor agonists positively affect bone metabolism and remodeling essential for spinal fusion, with liraglutide demonstrating the ability to reverse osteopenia in animal models, potentially explaining our observed improved outcomes [37].

This meta-analysis possesses several strengths. First, our meta-analysis represents the first comprehensive systematic review and meta-analysis examining GLP-1 receptor agonist effects on spinal fusion outcomes. The analysis encompasses a large number of participants, which is sufficient to provide evidence. The methodological quality of included studies was consistently high, with almost all included studies providing good quality. Our publications were exclusively from 2025, which reflects the importance of answering our research question in the current time, as the idea is novel and can provide valuable insights for future research. Most analyses demonstrated low statistical heterogeneity across the included studies, and we applied sensitivity analysis when available to resolve heterogeneity. Finally, we performed a subgroup analysis of patient diabetes status and surgical procedure type, enabling more personalized clinical decision-making regarding the use of GLP-1 in different scenarios.

Despite the stated strengths, several limitations warrant consideration. All included studies were retrospective observational studies. Some data may be overlapped due to the use of the same databases within the included studies, without any specifications for the included population within these studies. The definition of GLP-1 receptor agonist exposure varied in timelines between the studies, and some analyses were limited by small numbers of contributing studies, particularly for certain subgroup analyses where single studies provided data for specific comparisons.

Future research should prioritize well-designed RCTs to establish definitive causal relationships between GLP-1 receptor agonist use and spinal fusion outcomes. These studies should incorporate standardized outcome definitions, particularly for pseudoarthrosis assessment using consistent radiographic criteria and timing. Prospective studies with predetermined GLP-1 receptor agonist dosing protocols and administration timing would provide more precise therapeutic guidance. Mechanistic studies examining the biological pathways for both bone healing benefits and renal safety are required to optimize the use of the drug as an adjunct therapy.

## Conclusions

This represents the first meta-analysis examining GLP-1 receptor agonist therapy in spinal fusion, providing high-level evidence from a large population. Our findings demonstrate promising therapeutic potential with significant reductions in pseudoarthrosis rates at the 6-month and 12-month follow-up periods. Although overall 24-month results were non-significant, subgroup analysis demonstrated superiority for GLP-1 patients in lumbar fusion patients at this time point. However, our analysis revealed a concerning increase in acute kidney injury risk, contrasting with established renal protective effects in cardiovascular and diabetes trials. This discrepancy may reflect perioperative physiological stresses that alter the drug’s typical nephroprotective profile. However, the identified renal safety signal necessitates careful patient selection and monitoring. Future research should focus on well-designed RCTs investigating optimal dosing protocols, patient selection criteria, and the mechanistic basis for both bone healing benefits and unexpected renal complications.

## Appendices

Supplementary search strategy

**Table 4:** Detailed search strategy.

Database	Search query	Number of results
PubMed	(“Glucagon Like Peptide 1” OR “Glucagon-Like Peptide-1” OR “GLP 1” OR GLP-1 OR GLP1R OR Semaglutide OR Ozempic OR Wegovy OR Rybelsus OR Liraglutide OR Victoza OR Saxenda OR Dulaglutide OR Trulicity OR Exenatide OR Byetta OR Bydureon OR Lixisenatide OR Adlyxin OR Lyxumia OR Efpeglenatide OR Albiglutide OR Tirzepatide OR Mounjaro OR Zepbound OR Tanzeum OR Eperzan OR twincretin OR “incretin mimetic*”) AND (Fusion OR Spondylodes* OR Spondylosyndes* OR arthrodesis OR ACDF) AND (lumb* OR spin* OR cervi* OR thorac* OR vertebra* OR neck OR ACDF OR back OR Spondylodes* OR Spondylosyndes* OR arthrodesis)	26
WOS	ALL=((“Glucagon Like Peptide 1” OR “Glucagon-Like Peptide-1” OR “GLP 1” OR GLP-1 OR GLP1R OR Semaglutide OR Ozempic OR Wegovy OR Rybelsus OR Liraglutide OR Victoza OR Saxenda OR Dulaglutide OR Trulicity OR Exenatide OR Byetta OR Bydureon OR Lixisenatide OR Adlyxin OR Lyxumia OR Efpeglenatide OR Albiglutide OR Tirzepatide OR Mounjaro OR Zepbound OR Tanzeum OR Eperzan OR twincretin OR “incretin mimetic*”) AND (Fusion OR Spondylodes* OR Spondylosyndes* OR arthrodesis OR ACDF) AND (lumb* OR spin* OR cervi* OR thorac* OR vertebra* OR neck OR ACDF OR back OR Spondylodes* OR Spondylosyndes* OR arthrodesis))	16
Scopus	TITLE-ABS-KEY ( (“Glucagon Like Peptide 1” OR “Glucagon-Like Peptide-1” OR “GLP 1” OR GLP-1 OR GLP1R OR Semaglutide OR Ozempic OR Wegovy OR Rybelsus OR Liraglutide OR Victoza OR Saxenda OR Dulaglutide OR Trulicity OR Exenatide OR Byetta OR Bydureon OR Lixisenatide OR Adlyxin OR Lyxumia OR Efpeglenatide OR Albiglutide OR Tirzepatide OR Mounjaro OR Zepbound OR Tanzeum OR Eperzan OR twincretin OR “incretin mimetic*” ) AND ( Fusion OR Spondylodes* OR Spondylosyndes* OR arthrodesis OR ACDF ) AND ( lumb* OR spin* OR cervi* OR thorac* OR vertebra* OR neck OR ACDF OR back OR Spondylodes* OR Spondylosyndes* OR arthrodesis ) )	29
Cochrane	((“Glucagon Like Peptide 1” OR “Glucagon-Like Peptide-1” OR “GLP 1” OR GLP-1 OR GLP1R OR Semaglutide OR Ozempic OR Wegovy OR Rybelsus OR Liraglutide OR Victoza OR Saxenda OR Dulaglutide OR Trulicity OR Exenatide OR Byetta OR Bydureon OR Lixisenatide OR Adlyxin OR Lyxumia OR Efpeglenatide OR Albiglutide OR Tirzepatide OR Mounjaro OR Zepbound OR Tanzeum OR Eperzan OR twincretin OR “incretin mimetic*”) AND (Fusion OR Spondylodes* OR Spondylosyndes* OR arthrodesis OR ACDF) AND (lumb* OR spin* OR cervi* OR thorac* OR vertebra* OR neck OR ACDF OR back OR Spondylodes* OR Spondylosyndes* OR arthrodesis))	1
Embase	(‘glucagon like peptide 1’/exp OR ‘glucagon like peptide 1’ OR ‘glucagon-like peptide-1’/exp OR ‘glucagon-like peptide-1’ OR ‘glp 1’/exp OR ‘glp 1’ OR glp1r OR ‘semaglutide’/exp OR semaglutide OR ‘ozempic’/exp OR ozempic OR ‘wegovy’/exp OR wegovy OR ‘rybelsus’/exp OR rybelsus OR ‘liraglutide’/exp OR liraglutide OR ‘victoza’/exp OR victoza OR ‘saxenda’/exp OR saxenda OR ‘dulaglutide’/exp OR dulaglutide OR ‘trulicity’/exp OR trulicity OR ‘exenatide’/exp OR exenatide OR ‘byetta’/exp OR byetta OR ‘bydureon’/exp OR bydureon OR ‘lixisenatide’/exp OR lixisenatide OR ‘adlyxin’/exp OR adlyxin OR ‘lyxumia’/exp OR lyxumia OR ‘efpeglenatide’/exp OR efpeglenatide OR ‘albiglutide’/exp OR albiglutide OR ‘tirzepatide’/exp OR tirzepatide OR ‘mounjaro’/exp OR mounjaro OR ‘zepbound’/exp OR zepbound OR ‘tanzeum’/exp OR tanzeum OR ‘eperzan’/exp OR eperzan OR twincretin OR ‘incretin mimetic*’) AND (‘fusion’/exp OR fusion OR spondylodes* OR spondylosyndes* OR ‘arthrodesis’/exp OR arthrodesis OR acdf) AND (lumb* OR spin* OR cervi* OR thorac* OR vertebra* OR ‘neck’/exp OR neck OR acdf OR ‘back’/exp OR back OR spondylodes* OR spondylosyndes* OR ‘arthrodesis’/exp OR arthrodesis)	41
